# Supplementary material for: A binary interaction map between turnip mosaic virus and Arabidopsis thaliana proteomes
Source: Commun Biol. 2023 Jan 11;6:28. doi: 10.1038/s42003-023-04427-8 (PMC9834402; doi:10.1038/s42003-023-04427-8)
Supplement: Supplementary file 2 — Supplementary Material [file 42003_2023_4427_MOESM2_ESM.pdf]

Supplementary Materials for

A binary interaction map between turnip mosaic virus and *Arabidopsis thaliana* proteomes

Fernando Martínez[1,†], José L. Carrasco[1,†], Christina Toft[1], Julia Hillung[1], Silvia Giménez-Santamarina[2], Lynne Yenush[2], Guillermo Rodrigo[1,†,*], and Santiago F. Elena[1,3,*]

[1]Instituto de Biología Integrativa de Sistemas (I[2]SysBio), CSIC - Universitat de València, 46980 Paterna, Spain

[2]Instituto de Biología Molecular y Celular de Plantas (IBMCP), CSIC - Universitat Politècnica de València, 46022 València, Spain

[3]The Santa Fe Institute, Santa Fe NM87501, USA

[†]These authors contributed equally

[*]Correspondence: guillermo.rodrigo@csic.es (G.R.) and santiago.elena@csic.es (S.F.E.)

This PDF file includes:

Supplementary Figs. 1 to 5: figure referenced in Main text;

Supplementary Tables 1 to 3: tables referenced in Main text;

Supplementary References: referenced in Supplementary Table 3.

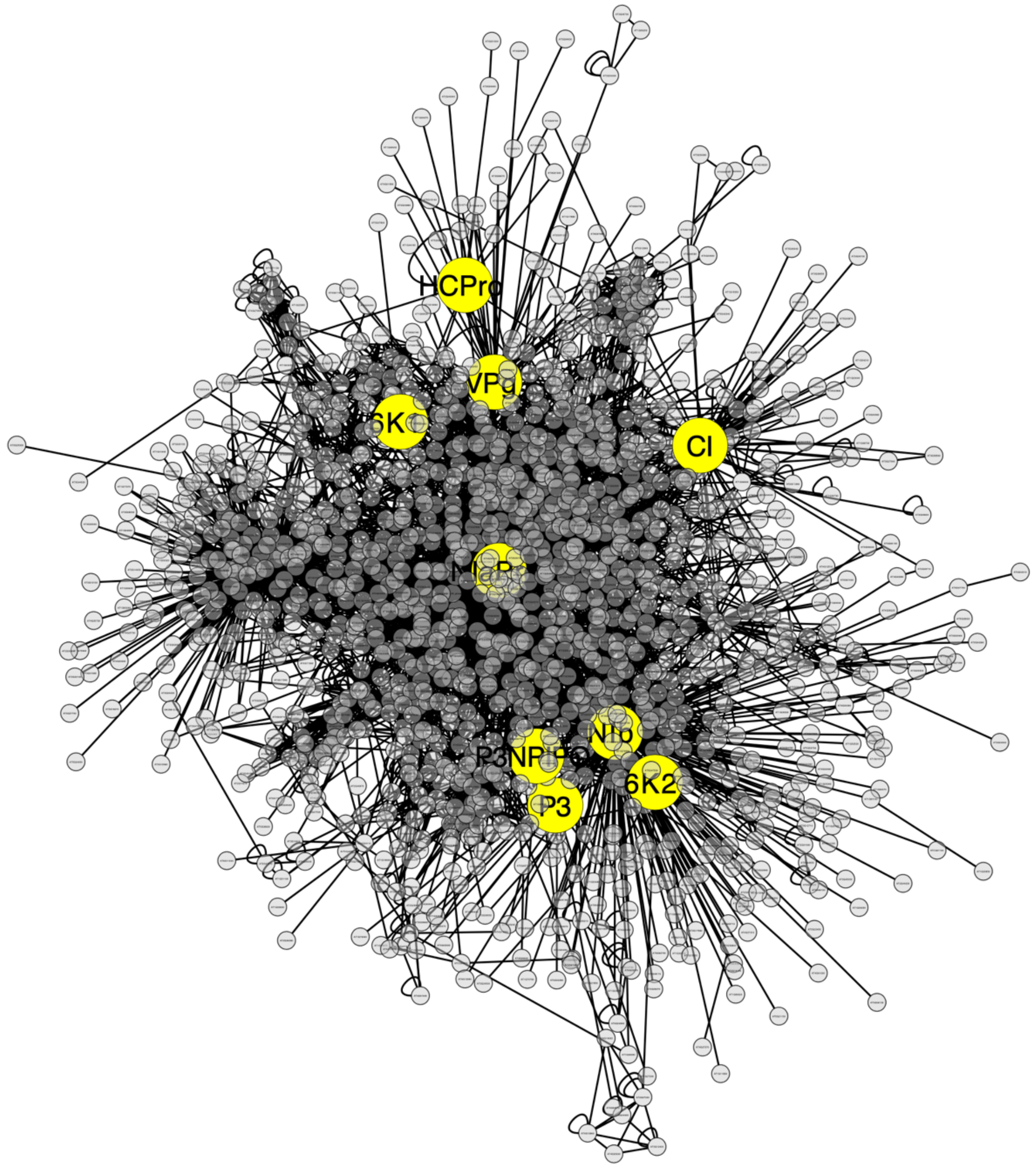

Supplementary Figure 1: Extended network of virus-host PPI that includes the one-step neighbors of the host proteins targeted by the virus are included. This extended network includes virus-virus, virus-host and also protein-protein interactions stablished between the viral targets and all their one-step neighbors in the AI-1_{MAIN} network. The different viral proteins are highlighted in yellow.

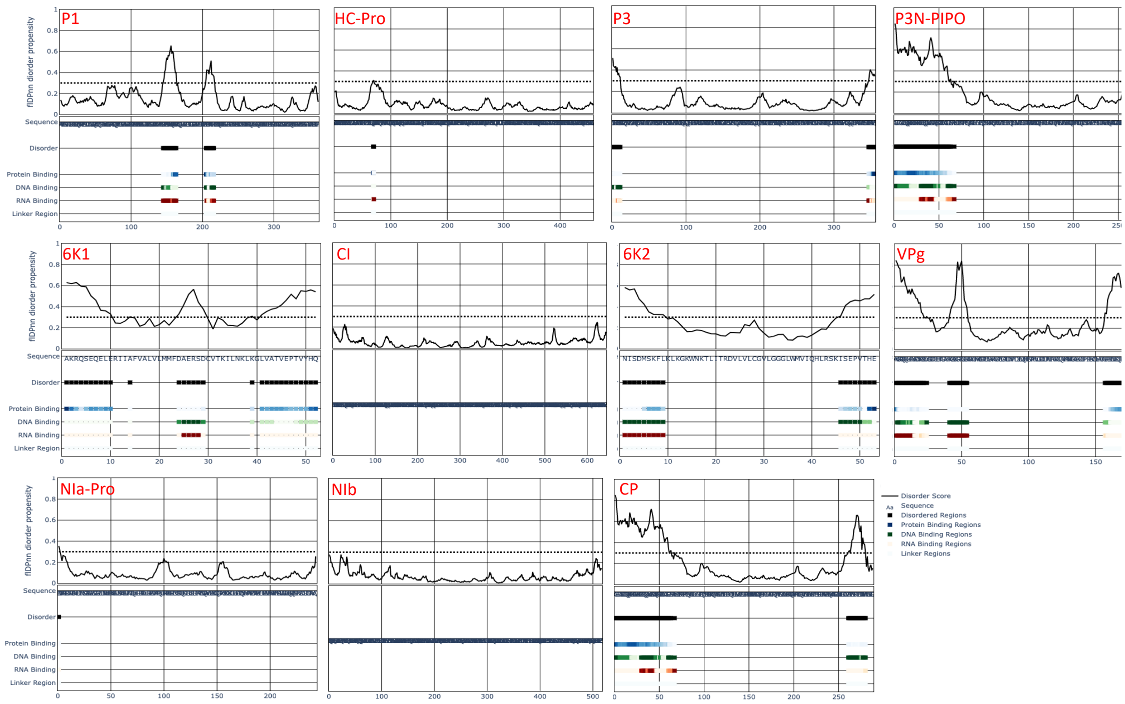

Supplementary Figure 2: Prediction of intrinsically disordered protein regions (IDPR) for the 11 TuMV proteins. Predictions were done using the deep neural network implemented in the fIDPnn server (https://biomine.cs.vcu.edu/servers/fIDPnn/). P1, P3N-PIPO, 6K1, 6K2, VPg, and CP show the more significant IDPR predictions.

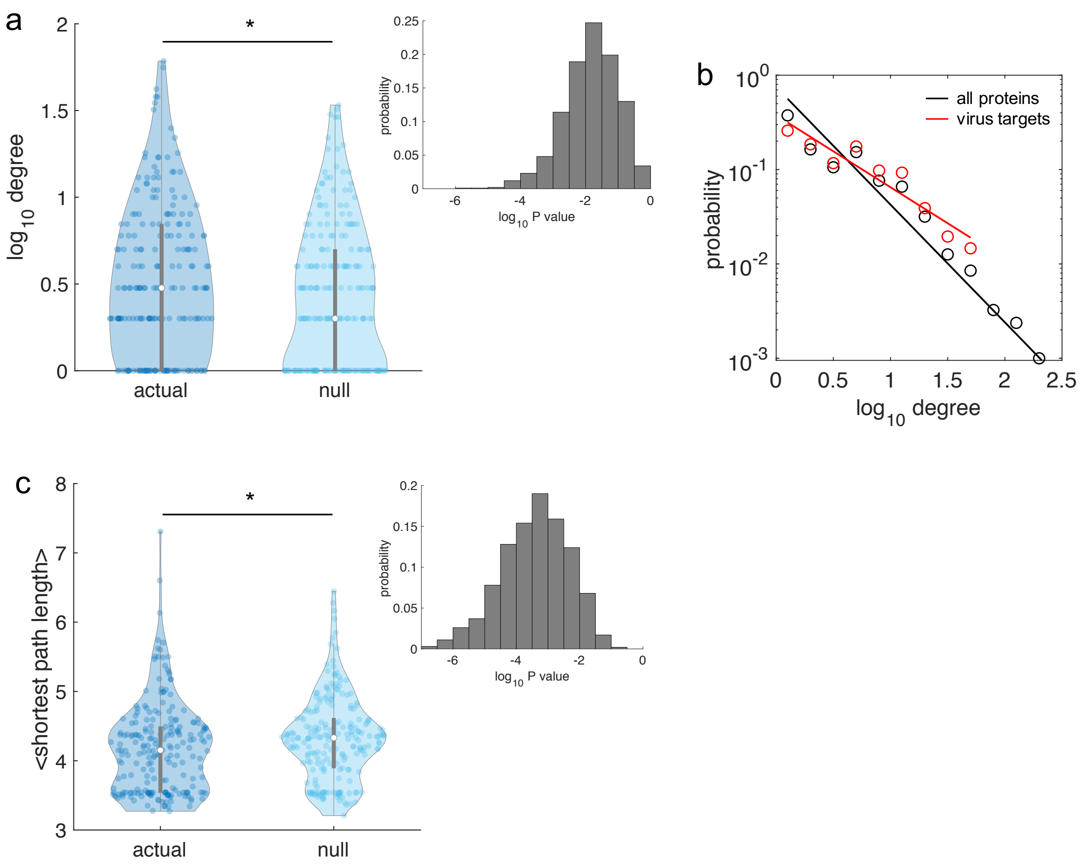

Supplementary Figure 3: Reanalysis of connectivity degree of TuMV targets excluding interactions collected from the literature. a) Comparison between the actual degree distribution (from virus targets) and a representative null distribution (from randomly picked genes). *Statistical significance (Mann-Whitney U test, $P < 0.05$). The inset shows the distribution of P values after 1000 random realizations, with geometric mean 0.0145. b) Probability distribution of the degree for only the virus targets (red) or all proteins in the host interactome (black). Points correspond to the data, whilst lines to the best-fitting power law probability ~ degree$^{-\gamma}$ ($\gamma = 0.763$ for virus targets and $\gamma = 1.247$ for all proteins). c) Comparison between the actual shortest path length distribution (from virus targets) and a representative null distribution (from randomly picked genes). *Statistical significance (Mann-Whitney U test, $P < 0.05$). The inset shows the distribution of P values after 1000 random realizations, with geometric mean 0.0040.

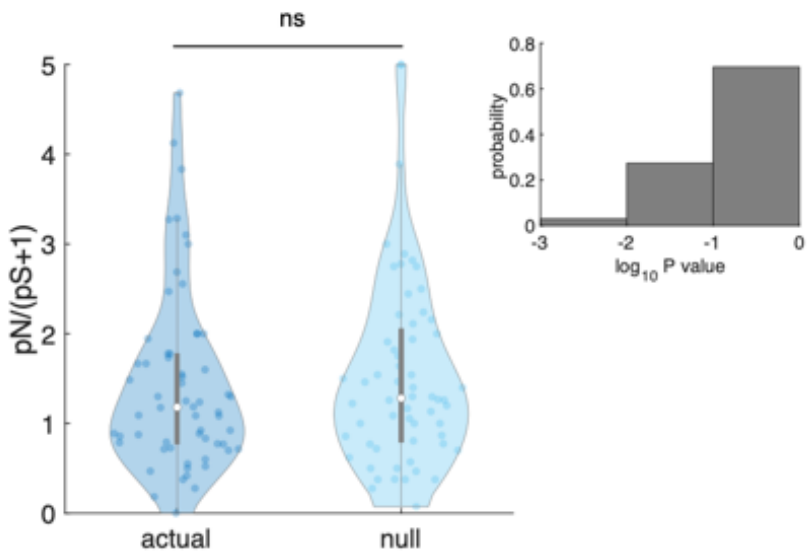

Supplementary Figure 4: Reanalysis of the evolutionarily conservation of TuMV-interacting host proteins filtering by high connectivity degree. Only for *A. thaliana* proteins with connectivity degree ≥ 5 in the AI-1_{MAIN} network, comparison between the actual p_N/p_S distribution and a representative null distribution (from randomly selected proteins). [ns]Statistically not significant (Mann-Whitney *U* test, $P > 0.05$). The inset shows the distribution of *P* values after 1000 random realizations, with geometric mean 0.16.

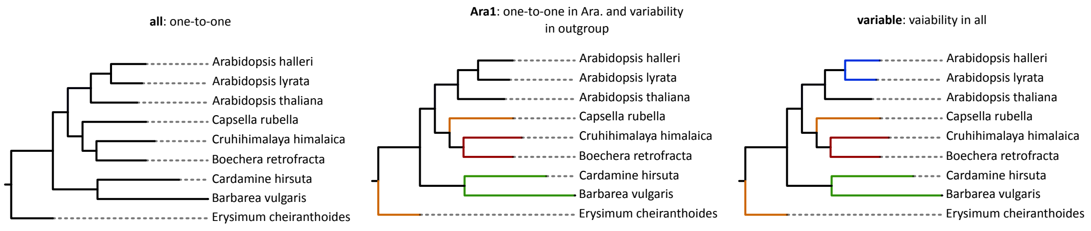

Supplementary Figure 5: Categories of orthologous groups in the ω evolutionary analysis. All: the orthologous groups containing a one-to-one relationship between all nine genomes. **Ara1:** the orthologous groups containing a one-to-one relationship within the Arabidopsis genus but allows for some pruning of the remaining six genomes and for lacking one of the genes in a colored clade (red and green clade). **Variable:** this is the most variable category which only needs to have a single gene from *A. thaliana*, the rest can be pruned and lack one of the genes in a colored clade (blue, red and green clade). Green, red and blue branch pairs: allows for lacking one of the genes within the clade and pruning, black branches: only one gene within the orthologous group, orange: allows for pruning of duplicated genes.

Supplementary Table 1: Mutant *A. thaliana* lines used to validate the predicted interactors.

Gene	AGI code	Mutant line
CNX1	*AT5G61790*	SALK_083600C
NPR1-1	*AT1G64280*	EMS mutant
OBE1-1	*AT3G07780*	SALK_075710C
PCaP1	*AT4G20260*	SALK_022955C
PIP1;3	*AT1G01620*	SALK_051107C
RP40	*AT1G72370*	SALK_119138
SCE1	*AT3G57870*	SALK_006164
SGS	*AT1T30070*	GK-106C03
SUMO3	*AT5T55170*	GK-368C03
TGA1	*AT5T65210*	*tga1-1* (SALK_028212)

Supplementary Table 2: List of primers used to amplify each tested *A. thaliana* mutant genotype.

Gene	T-DNA line	LP Sequence	RP Sequence
RP40	SALK_119138	GATCCAATCAAAGGAGTTCGAG	TATGGAATGGTAGGTGGAGACC
SCE1	SALK_006164	GCAGAGTTCAGTTTCAATGTCG	TTCTGTTTGACAAATGTTTGCC
SGS	GK-106C03	CTCGCCATAATCGTTTCCTTAC	GTCCTTTTAAAGGATCTGCTGC
SUMO3	GK-368C03	ACCGTTTGATTTTGATCAAACC	TACAATGATTATGAGCCGTTCG

Supplementary Table 3: List of plant species used for the evolutionary analyses.

Species	Reference to genome
Arabidopsis halleri L.	1
Arabidopsis lyrata L.	2
Arabidopsis thaliana (L.) Heyhn	3
Barbarea vulgaris W.T. Aiton	4
Boechera retrofracta (Graham) Á. Love & D. Love	5
Capsella rubella Reut.	6
Cardamine hirsuta L.	7
Crucihimalaya himalaica (Edgew) Al-Shehbaz, O'Kane & R.A. Price	8
Erysimum cheiranthoides L.	9

Supplementary References

1. Briskine, R.V., Paape, T., Shimizu-Inatsugi, R., Nishiyama, T., Akama, S., Sese, J., *et al*. Genome assembly and annotation of *Arabidopsis halleri*, a model for heavy metal hyperaccumulation and evolutionary ecology. *Mol. Ecol. Resour.* **17**, 1025-1036 (2017).
2. Hu, T.T., Pattyn, P., Bakker, E.G., Cao, J., Cheng, J.F., Clark, R.M., *et al*. The *Arabidopsis lyrata* genome sequence and the basis of rapid genome size change. *Nat. Genet*. **43**, 476-481 (2011).
3. Arabidopsis Genome Initiative. Analysis of the genome sequence of the flowering plant *Arabidopsis thaliana*. *Nature* **408**, 796-815 (2000).
4. Byrne, S.L., Erthmann, P.Ø., Agerbirk, N., Bak, S., Hauser, T.P., Nagy, I., *et al*. The genome sequence of *Barbarea vulgaris* facilitates the study of ecological biochemistry. *Sci. Rep*. **7**, 40728 (2017).
5. Kliver, S., Rayko, M., Komissarov, A., Bakin, E., Zhernakova, D., Prasad, K., *et al*. Assembly of the *Boechera retrofracta* genome and evolutionary analysis of apomixis-associated genes. *Genes* **9**, 185 (2018).
6. Slotte, T., Hazzouri, K.M., Ågren, J.A., Koenig, D., Maumus, F., Guo, Y.L., *et al*. The *Capsella rubella* genome and the genomic consequences of rapid mating system evolution. *Nat. Genet.* **45**, 831-835(2013).
7. Gan, X., Hay, A., Kwantes, M., Haberer, G., Hallab, A., Dello, R., *et al*. The *Cardamine hirsuta* genome offers insight into the evolution of morphological diversity. *Nat. Plants* **2**, 16167 (2016).
8. Zhang, T., Qiao, Q., Novikova, P.Y., Wang, Q., Yue, J., Guan, Y., *et al*. Genome of *Crucihimalaya himalaica*, a close relative of *Arabidopsis*, shows ecological adaptation to high altitude. *Proc. Natl. Acad. Sci. USA* **116**, 7137-7146 (2019).
9. Züst, T., Strickler, S.R., Powell, A.F., Mabry, M.E., An, H., Mirzaei, M., *et al*. Independent evolution of ancestral and novel defenses in a genus of toxic plants (*Erysimum*, *Brassicaceae*). *eLife* **9**, e51712 (2020).
