# Supplementary material for: A binary interaction map between turnip mosaic virus and Arabidopsis thaliana proteomes
Source: Commun Biol. 2023 Jan 11;6:28. doi: 10.1038/s42003-023-04427-8 (PMC9834402; doi:10.1038/s42003-023-04427-8)
Supplement: Supplementary file 3 — Description of Additional Supplementary Files [file 42003_2023_4427_MOESM3_ESM.pdf]

Description of Additional Supplementary Files

File name: Supplementary Data 1
Description: The list of all host interactors identified in this work by HT-Y2H screening.

File name: Supplementary Data 2
Description: A literature-curated list of all physical potyvirus plant interactions already described.

File name: Supplementary Data 3
Description: AI-1MAIN network model used in this study.

File name: Supplementary Data 4
Description: Results of the different selection analyses (pN/pS and ω) for each A. thaliana gene.

File name: Supplementary Data 5
Description: All primers used to generate the different genetic constructions of this work.
